# Editorial: Subclassification of AUS/FLUS category for thyroid nodules: trials and evidence-based clinical management

**DOI:** 10.3389/fendo.2023.1209776

**Published:** 2023-06-13

**Authors:** Taner Bayraktaroğlu, Figen Barut, Güldeniz Karadeniz Çakmak

**Affiliations:** ^1^ Divison of Endocrinology and Metabolism, Department of Internal Medicine, Zonguldak, Türkiye; ^2^ Department of Pathology, Faculty of Medicine, Zonguldak, Türkiye; ^3^ Department of General Surgery, Faculty of Medicine, Zonguldak Bulent Ecevit University, Zonguldak, Türkiye

**Keywords:** Bethesda System for Reporting Thyroid Cytopathology, Atypia of Undetermined Significance/Follicular Lesion of Undetermined Significance, AUS/FLUS, Thyroid Nodules, Fine Needle Aspiration Cytology

## Introduction

This Research Topic aims to include original research articles, reviews, systematic reviews, meta-analyses, on themes related to the “atypia of undetermined significance/follicular lesion of undetermined significance” (AUS/FLUS) category regarding Bethesda System for Reporting Thyroid Cytopathology (BSRTC) (1–4). The fırst article of this Research Topic concluded that the malignancy rate of thyroid nodules with AUS/FLUS cytology was comparable irrespective of the presence of underlying chronic lymphocytic thyroiditis (CLT) (Cho et al.). In an other study, risk stratification w recommended for patients with Bethesda category III (AUS/FLUS) nodules with a size under 1 cm. Nodules with a size over 1 cm harboring WT-BRAF or those under 1 cm harboring BRAF V600E mutation was regarded as moderate risk, and molecular testing should be recommended. However, those with a size over 1 cm harboring BRAF V600E mutation should be regarded as high risk, and a diagnostic surgery should be recommended (Zha et al.) In addition, malignant disease was common in Bethesda category III nodules, and surgical treatment was strongly indicated in the presence of male sex, aspect ratio>1, microcalcification, and BRAFV600E mutation (Liu et al.).

Fine needle aspiration cytology is the gold standard method for the differential diagnosis of thyroid nodules, though 25–30% of which are classified as indeterminate in categories III or IV of the Bethesda System for Reporting Thyroid Cytopathology (1, 2). The reported confirmation rate of the malignancy in surgical specimens is up to 40% for indeterminate nodules which means most of the patients may be subjected to unnecessary surgical interventions (5).

**Figure 1:**
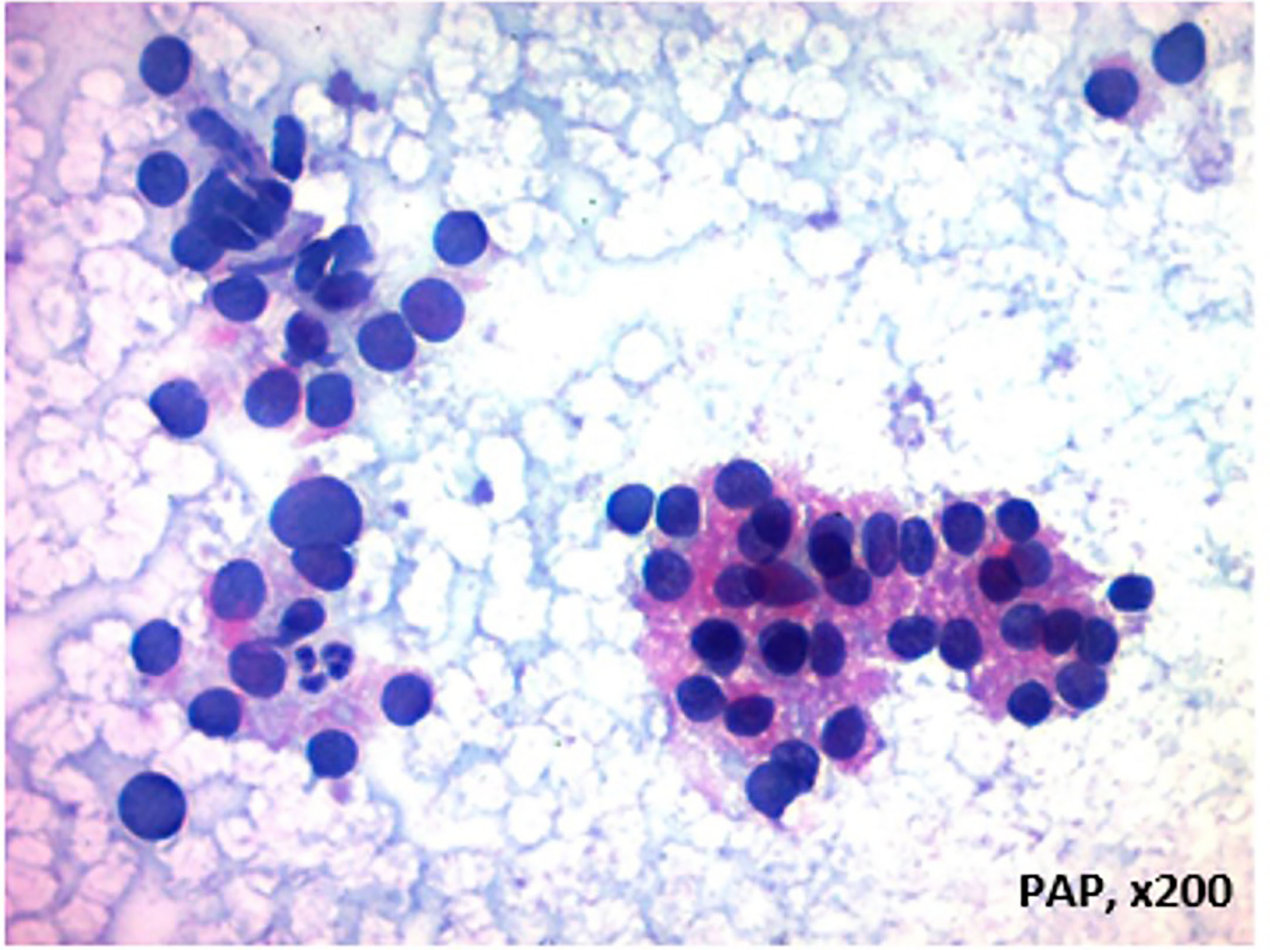
Thyroid epithelial cells that form a three-dimensional cell layer with few microfollicule-like structures and exhibit hyperchromasia and pleomorphism in some areas, AUS/Bethesda III Category author (It was used from FB's archive and permisssion).

Bethesda category III namely, atypia of undetermined significance/follicular lesion of undetermined significance (AUS/FLUS) represents the gray zone in thyroid cytopathology for which the decision making process might be even harder either for patient or the physician. (**Figure 1**). The terms “indeterminate” and “atypical” may be interpreted differently by different medical professionals and institutions, leading to confusion in diagnostic and therapeutic management of thyroid nodules. This can lead to variation in patient care and outcomes. Therefore, it’s important to have standardized guidelines and criteria for the interpretation and management of thyroid nodules to ensure consistency and accuracy in diagnosis and treatment (6, 7, Liu et al.; Zha et al.).

## The risk of malignancy associated with AUS/FLUS

The risk of malignancy (ROM) associated with different subgroups remains unresolved; ROM for AUS/FLUS varies greatly among medical centers (8, 9, Cho et al.; Zha et al.). Subclassification of AUS/FLUS might be helpful in identifying nodules with a high ROM in this category and improving the management of such nodules. The AUS subcategory indicates a higher risk of malignancy than the FLUS subcategory. To resolve some of these problems, recent studies of the malignancy risk in AUS/FLUS suggest a two- tier subclassification of the AUS/FLUS category (2, 5):

1-Low cellularity with predominant microfollicular architecture and absence of colloid,
2-Nuclear features not characteristic of benign lesions (nuclear atypia) attributable to strict criteria for the evaluation of cancer probability.

## Management AUS/FLUS - trials and evidence-based outcomes

The rates of malignancy among this category have varied widely, ranging from 6 – 48% among selected subsets of the patient’s undergoing surgery. In this context, ultrasound (US) classifications, allowing a better classification of malignant nodules, have developed worldwide. Among them, the European Thyroid Association guidelines introduced in 2017, a US classification for thyroid nodules, the EU-TIRADS, which was confirmed to be a highly sensitive system and is widely used among European Countries (10, 11). The American College of Radiology (ACR) recommended the Thyroid Imaging Reporting and Data System (TI-RADS) as a classification system. ACR TI-RADS predicts the probability of malignancy in thyroid nodules using a scoring system (TR1-TR5) based on multiple ultrasound characteristics and nodule size (12). Various scoring systems have been developed to combine ultrasound findings with clinical information to more accurately predict the risk of malignancy in thyroid nodules (13, 14). Moreover, upon the improved knowledge on the genetic characterization of thyroid cancer (TC), several studies focused on the molecular evaluation of nodules to optimize the management of cytological indeterminate nodules (15–19), as recommended in current guidelines (20, 21).

The available molecular diagnostic tools are classified into two different categories: ‘rule-out’ methods, which have the purpose to reduce the avoidable treatment of benign nodules, and ‘rule-in’ approaches that aim to optimize surgical management (total thyroidectomy or diagnostic lobectomy). Among the rule-in methods, the last version of ThyroSeq (TSv3) can identify more than 12,000 hotspot mutations and more than 120 fusions, while the rule out Afrma Genomic Sequencing Classifier (GSC) analyzes the expression profiles of 1115 genes and detects single nucleotide variants and fusions. Additional approaches for molecular testing include the analysis of miRNAs expression (22). Although both GSC and TSv3 have been demonstrated to be considerably more cost-effective than diagnostic lobectomy (23). However, the main limitation of these molecular tests is their high cost (25) which largely restricts their application in clinical practice, especially in European Countries.

Customized 5-7 genes rule-in panels have been set up to analyze the most frequent genetic alterations found in TC (22). A developed PTC-MA assay able to evaluate in an extremely cost-effective manner, a total of 24 genetic alterations including point mutations and fusions frequently found in TC (25–27). Afirma, ThyroSeq, and ThyGenX/ThyGeNEXT with ThyraMIR are covered by Medicare and many health insurance plans, including BlueCross/BlueShield in the United States, (28, 29). In the 2017 European Thyroid Association guideline (27), the authors concluded that there may be benefit to considering genetic panels that include BRAF, RET/PTC, PZX8/PPARG, NTRK, and RAS mutations for nodules of indeterminate cytology, but they did not recommend the routine use of the Afirma GEC to exclude malignancies because validation studies in the form of long-term outcome data are lacking (28).

The thyroid risk score (TRS) system was developed to further enhance the sensitivity and specificity of the molecular tool. It is based on the combination of clinical, ultrasound, cytological, and molecular criteria (**Table 1**) (27).

**Table 1:** Thyroid Scoring Systems.

THYROID SCORING SYSTEMS				
Cytology (Bethesda)*	III	–	IV	–
**Nodule Size (mm)**	≤10	10 - 20	20 – 40	≥ 40
EU-TIRADS**	2	3	4	5 and/or LFN Mt.
**SCORE**	1	1,5	2	2,5
**Molecular data**	Wild Type	Ret/PTC	EIFA1X	BRAF
		PAX8/PPARƔ		N-TRK
		N-/K-/H-RAS		pTERT
				AKT
				PIK3CA
**Thyroid Risk Score**	≤ 4 LIKELY BENIGN	>4 ≤6 LOW SUSPICION	>6 ≤ 8 INTERMEDIATE SUSPICION	> 8 High SUSPICION

*^2^;*^10^.

A diﬀerent suspicion for malignancy was arbitrarily assigned to the diﬀerent scores.

The pre-surgical thyroid risk score (TRS) including clinical, ultrasonography, cytological and molecular features of indeterminate thyroid nodules adapted from 27.

**European Thyroid association guidelines for ultrasound malignancy risk stratification of thyroid nodules in adults: the EU-TIRADS (11).

The indication to surgery was based on the following parameters (27):

– Risk score ≥6
– Large goiters with compression independently from the risk score
– Presence of at least one genetic mutation. It is worth noting that all mutated cases were ultimately found to harbor a risk score ≥6.

Though 14 point mutations and 11 fusions have been tested, only seven genetic alterations were found (in the HRAS, NRAS, BRAF, TERT, EIF1AX, PAX8, RET genes), which are likely to be the more frequently represented in the indeterminate samples. The rate of follicular thyroid carcinomas (FTCs) included (8%) is even higher than that reported in large validation case series (30–32).

## Perspectives

This Research Topic perspectives include original research articles, reviews, meta-analyses, on themes related to the AUS/FLUS. Thyroid nodules with indeterminate cytology are one of the most important challenges for the endocrinologist and surgeon. The definitive diagnosis of these nodules requires a histological evaluation after surgery, which represents an overtreatment, and a considerable waste of resources for the national healthcare systems and patients facing potential complications in most benign cases (60–80% of total indeterminate nodules). Ultrasound risk evaluation and molecular testing have been shown to be useful tools in guiding the clinical management of thyroid nodules with indeterminate cytology. However, ultrasound risk evaluation may be limited in its specificity, and molecular testing may be limited by its high cost. Therefore, these tools should be used in conjunction with other clinical and laboratory evaluations to optimize patient care. These parameters have been almost always considered as separate tools for the presurgical differential diagnosis of indeterminate nodules.

A combined score including different parameters, previously validated as diagnostic tools, generated to increase the preoperative accuracy is important. In particular, ‘thyroid risk score’ (TRS), ACR TI-RADS prediction system, the EU-TIRADS scoring and the Bethesda classification, characterized by a high sensitivity (12, 33, 34).

We hope that the reader will find in this Research Topic a useful reference for the current literature in the emerging field of BETHESDA III category as AUS/FLUS.

In conclusion, this Research Topic provides multidisciplinary;

• Bethesda System for Reporting Thyroid Cytopathology (BSRTC)
• The “atypia of undetermined significance/follicular lesion of undetermined significance” (AUS/FLUS) category
• Diagnostic categories and evidence-based clinical management.
• Presurgical differential diagnosis of indeterminate nodules
• Thyroid risk score’ (TRS),
• The EU-TIRADS scoring, Ultrasound risk evaluation
• The risk of malignancy (ROM) for AUS/FLUS
• Validated as diagnostic tools, generated to increase the preoperative accuracy
• BRAF molecular testing for the stratification of ROM into high-risk and low-risk AUS cases;
• Morphological subcategorization, based on atypia qualifiers and molecular testing, potential improvement of malignancy risk stratification of AUS/FLUS patients;
• Different qualifiers of AUS/FLUS thyroid FNA e.g. BRAF, RAS, RET/PTC, and PAX8/PPARg alterations
• Hybrid molecular and morphological subcategorization systems in improving the malignancy risk stratification of thyroid FNA samples diagnosed as AUS/FLUS.

We welcome original research, reviews, perspective, and thought-provoking opinion to this Research Topic.

## Author contributions

All authors listed have made a substantial, direct, and intellectual contribution to the work and approved it for publication. TB, FB and GKÇ contributed to conception and design of the article. TB wrote the first draft of the manuscript. FB prepared and added a AUS/FLUS Figure in the text. All authors contributed to manuscript revision, read, and approved the submitted version.
